# Another Differentiated Look at Emotions: A Replication Study on Associations Between Gaze Behavior During the Processing of Affective Videos and Emotional Granularity

**DOI:** 10.1111/sjop.70112

**Published:** 2026-05-15

**Authors:** Jonas Potthoff, Albert Wabnegger, Anne Schienle

**Affiliations:** ^1^ Department of Psychology University of Graz Graz Austria

**Keywords:** emotion differentiation, emotional granularity, eye‐tracking, replication, visual attention

## Abstract

People with high emotional granularity (EG; the capacity to differentiate among discrete emotional states) tend to employ more effective emotion regulation strategies during negative affective states. Recent eye‐tracking evidence suggests that EG is associated with the frequency of revisits to negative emotional stimuli. The current study aimed to replicate this finding, however, using more complex, dynamic distractors to test whether avoidance occurs when competing content is more engaging and if the link between EG and revisits could be replicated. Participants (*n* = 160) viewed emotional videos and subsequently described their affect. The affect‐describing adjectives were used to calculate a performance‐based EG index. Higher performance‐based EG correlated with longer total fixation duration on affective videos. Higher self‐reported EG was associated with a lower number of gaze revisits of affective videos. The present replication study highlights the importance of considering the complexity of the visual environment when interpreting attentional indicators of emotion regulation. In the more complex visual environment, performance‐based EG may be linked to sustained engagement with emotional content while higher self‐reported EG may reduce the subjective need to revisit affect elicitors.

## Keypoints

- Replication study on emotional granularity and gaze behavior during visual affect processing using dynamic distractors with increased ecological validity.
- Performance‐based emotional granularity predicted longer gaze on emotional videos.
- Self‐reported emotional granularity predicted fewer gaze revisits to emotional videos.
- No correlation between performance‐based and self‐reported emotional granularity.

## 1 Introduction

Emotional granularity (EG) is the ability to distinguish between subtle variations of similar emotions (Smidt and Suvak 2015). Higher EG is associated with a reduced tendency to rely on nonspecific emotion regulation strategies, such as avoidance and distraction, which tend to be less effective in the long term (Brown et al. 2021). There is consensus that a higher EG is beneficial for mental health because high EG is linked to the selection of either situation‐specific (Brown et al. 2021) or more efficient emotion regulation strategies (Fugate et al. 2025; Kalokerinos et al. 2019). It has also been reported that EG can be trained to improve well‐being (Hoemann et al. 2021).

However, EG can be assessed in multiple ways, and different measures may relate differently to emotion regulation strategies. One common approach involves using questionnaires (Kang and Shaver 2004), which rely on the ability to reflect on the own capacity to differentiate between affective nuances. As such, self‐reported EG is likely biased depending on peoples' confidence in their abilities or how important EG is for their self‐concept (Robinson and Clore 2002; Thompson et al. 2021). A potentially less biased assessment involves performance‐based indices. Ottenstein and Lischetzke (2020) proposed a specificity index (SI), defined as the ratio of specific adjectives (e.g., “enraged”) to unspecific ones (e.g., “bad”) used to describe current affective states. The SI can be applied in both ecological momentary assessment (Ottenstein et al. 2024; Wabnegger et al. 2024) and laboratory research (Potthoff et al. 2023), enabling EG assessment without requiring recall of past emotional experiences.

Not only self‐reported EG but also self‐reported use of emotion regulation strategies may be biased by recall biases or people's' over‐ or underestimation of their own emotion regulation abilities. Psychophysiological measures like eye‐tracking can help assess indicators of emotion regulation strategies like avoidance behaviors more accurately and less biased than self‐report measures and may contribute insights into emotion regulation strategies which are not necessarily consciously perceived. A study by Potthoff et al. (2023) examined whether self‐reported EG (Kang and Shaver 2004) or performance‐based EG (Ottenstein and Lischetzke 2020) is associated with gaze avoidance of affect‐eliciting video clips (Blechert et al. 2013). Contrary to expectations, lower EG was not linked to gaze avoidance of negative videos. Instead, people with higher EG showed more frequent visual revisits to negative affect‐eliciting videos, suggesting an exploratory engagement of people who differentiate between their negative emotions in a more nuanced fashion.

The present study aimed to replicate a previous eye‐tracking study (Potthoff et al. 2023) with a key modification: instead of using static images of office supplies as neutral distractors being displayed alongside emotion‐eliciting videos, dynamic videos were presented. The prior study used static distractors (i.e., images of office supply), which tend to be less engaging than dynamic videos (Simons et al. 2000). By increasing the salience of the competing stimulus, the dynamic distractors (i.e., videos) in the present study create a more complex visual stimulation, better reflecting real‐world visual environments. We investigated whether the previously observed relationship between EG and visual attention to negative affective stimuli holds when the distractor is more engaging and visually more similar to the emotion eliciting stimulus. Furthermore, the present replication study of Potthoff et al. (2023) investigates whether gaze avoidance is associated with EG if the visual environment is more complex than in the previous study in which EG was not associated with gaze avoidance of negative affective stimuli.

In the current study, participants viewed positive and negative emotional videos alongside video distractors and subsequently described their emotions using adjectives. Performance‐based EG was assessed via the SI (proportion of specific adjectives; Ottenstein and Lischetzke 2020) and self‐reported EG via a questionnaire concerned with emotion differentiation (Kang and Shaver 2004). We hypothesized associations between EG and (1) the gaze duration on emotional videos (indicator of gaze avoidance) as well as with (2) the number of revisits on these videos (indicator of exploratory engagement). The study was preregistered in the Open Science Framework (OSF: https://osf.io/je3ar/).

## 2 Materials and Methods

### 2.1 Sample

Out of 160 participants with normal or corrected‐to‐normal vision (compare Potthoff et al. 2023), eye‐tracking data were successfully recorded for 151 participants (*n*_female_ = 90, *n*_male_ = 60, *n*_nonbinary_ = 1; age: *M* = 25.2 years, SD = 7.0 years). The majority of participants were university students (*n* = 117), with 93 having graduated from high school and 46 having finished at least an undergraduate degree. The study was conducted in German, and all participants either stated they were native German speakers or reported having German language proficiency at a native level. The study received approval from the university's ethics committee (ethics approval code: GZ. 39/69/63 ex 2022/23) and was conducted in accordance with the Declaration of Helsinki. All participants provided informed consent, and the study was conducted in accordance with the Declaration of Helsinki. University students received course credits for their participation. Other participants received no compensation.

### 2.2 Stimuli

Participants viewed 12 emotion‐eliciting videos from the *E.Vids* set (Blechert et al. 2013), in which an actor delivered either positive feedback (e.g., “One can really count on you.”; 6 trials) or negative feedback (e.g., “I can't bear you.”; 6 trials). Each video lasted 3 s, followed by a 3‐s still image of the final frame (compare: Potthoff et al. 2023).^1^ Each actor appeared exclusively in either a positive or a negative video, counterbalanced across participants.

The *E.Vids* were displayed on either the left or right side of the screen, while a distractor video of a bird was shown on the opposite side. The bird video was dynamic for the first 3 s, followed by a 3‐s still frame, allowing participants to shift their gaze between the affective stimulus and the alternative. All videos were cropped to a square format (800 × 800 px).

Video placement (left vs. right) and which actor provided positive/negative feedback was counterbalanced across four parallel versions of the paradigm, with participants randomly assigned to one version. The trial order was randomized for each participant. Participants were instructed to imagine that they were personally receiving the feedback presented in the videos (compare Wiggert et al. 2015). Before each trial, a fixation cross had to be fixated for 500 ms consecutively.

### 2.3 Self‐Reports (Ratings, Questionnaires) and Tests

#### 2.3.1 Trial Ratings

After each trial, participants described their experienced affect verbally with as many adjectives as they liked within 30 s. Subsequently, they reported the valence of their current emotional state on the 9‐point scale (1: most negative; 9: most positive) of the Self‐Assessment Manikin (SAM; Bynion and Feldner 2017). The procedure of trials is summarized in Figure 1.

**FIGURE 1:**
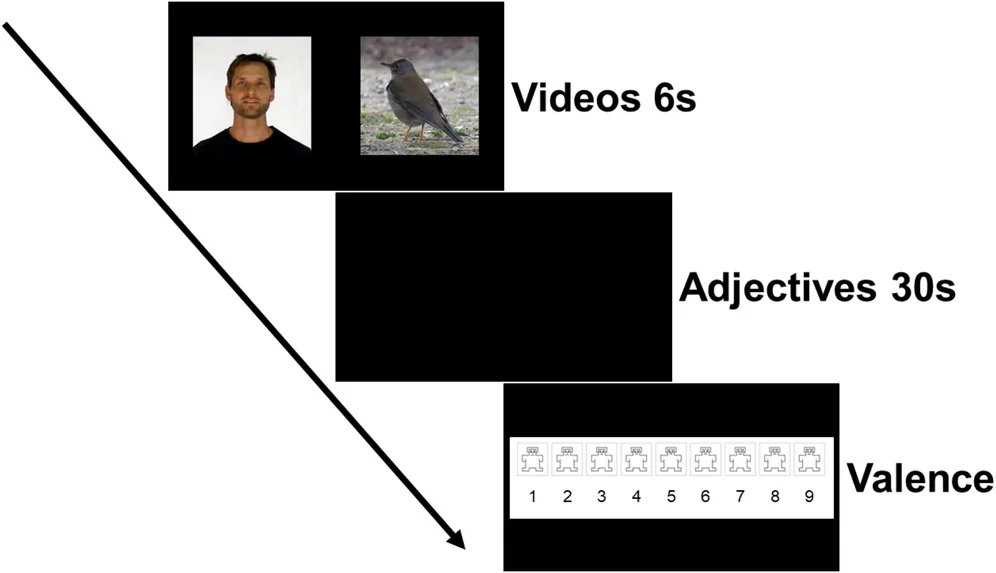
Experimental trial: Affect‐inducing videos were presented (6 s) followed by a blank screen (30 s), during which participants described their affective state via adjectives (assessment of performance‐based EG). Subsequently, the valence rating was completed (nine‐point self‐assessment manikin).

#### 2.3.2 Performance‐Based Emotional Granularity

A specificity index (SI) as a performance‐based indicator of EG was calculated separately for positive and negative trials based on the adjectives used to describe experienced emotions (e.g., “delighted” after positive feedback or “angry” after negative feedback) as suggested by Ottenstein and Lischetzke (2020). For each participant and trial, the number of specific adjectives was divided by the total number of affect‐describing adjectives. Three independent raters classified each adjective as either specific [e.g., “hurt” (German “verletzt”)] or unspecific [e.g., “bad” (German “schlecht”)]. We used specificity ratings for adjectives from Potthoff et al. (2023) and rated only those adjectives that had not been previously evaluated (*n* = 982 adjectives: 781 from Potthoff et al. 2023; 201 new adjectives). All three raters coded adjectives either as specific or unspecific (specific coded as 1, unspecific coded as 0; inter‐rater consistency: Fleiss kappa *κ* = 0.69; Shrout and Fleiss 1979). If not all three raters came to the same conclusion [9.35% of cases, e.g., “glücklich” (German for ~ “happy”)], the specificity was defined by the average rating. For example, if two raters coded an adjective as specific (i.e., 1) and one rater coded an adjective as unspecific (i.e., 0), the specificity of this adjective was two thirds (i.e., 2/3). All three raters were German native speakers and Psychologists who were instructed to rate adjectives as specific, if they were referring to a distinct affective state vs. general emotional tone or valence [e.g., “good” (German “gut”)]. A jamovi file with all adjectives and calculations of interrater reliability is provided in the OSF‐project: https://osf.io/ra7ku/.

#### 2.3.3 Self‐Reported Emotional Granularity

The differentiation scale of the Range and Differentiation of Emotional Experience Scale (RDEES; Kang and Shaver 2004) was used to assess self‐reported EG. This subscale has seven items (e.g., “I am aware of the subtle differences between feelings I have.”), rated on a 5‐point Likert scale ranging from 1 (“does not describe me very well”) to 5 (“describes me very well”). The scale demonstrated excellent internal consistency (Cronbach's *α* = 0.90).

#### 2.3.4 Further Questionnaires

For exploratory analyses we assessed the fear of negative evaluation (Reichenberger et al. 2016) and verbal fluency via an animal‐listing task (Tombaugh 1999). We assessed the demographic data age, gender, occupation, and education of participants.

### 2.4 Eye‐Tracking and Gaze Data Analysis

Following a 9‐point calibration, binocular gaze data were recorded using an *SMI RED250mobile* eye tracker (sampling rate: 250 Hz). Both eyes were calibrated, but only data from the eye with the more precise calibration were analyzed. Hence, if the accuracy for at least one eye was better than 0.5° of visual angle, the calibration was considered successful; otherwise, a recalibration was conducted. The average accuracy of the more accurately calibrated eye was *M* = 0.47° (SD = 0.19°). A chin rest minimized head movements and ensured a constant viewing distance of ~60 cm. The paradigm was conducted with *SMI Experiment Center* (version 3.6.53), and eye‐tracking data were exported using *SMI BeGaze* (version 3.6.52). Gaze events, including saccades and fixations, were defined using the default thresholds of *SMI BeGaze*: a saccade velocity threshold of 40°/s and a minimum fixation duration of 50 ms without a dispersion threshold. Blinks were defined as instances where the pupil diameter was zero (i.e., no pupil detected). Only fixation data were analyzed, while blinks and saccades were excluded from further data processing.

Eye‐tracking variables were calculated following Potthoff et al. (2023): (1) the percentage of total fixation duration spent on the *E.Vid* (and not on the distractor video) and (2) the number of revisits to the *E.Vid*, defined as instances where participants fixated on the affective video after conducting at least one fixation outside the *E.Vid* area of interest. Both videos and their corresponding AOIs had a size of 800 × 800 pixels. For five of the 1812 trials, it was not possible to calculate the total fixation duration percentage due to missing data (< 1% lost trials). No interpolation was conducted for these trials.

### 2.5 Statistical Analysis

The analysis approach followed that of Potthoff et al. (2023): *T*‐tests were computed to investigate whether the positive and negative trials elicited the intended valence of affect (manipulation check). Pearson correlations with 95% bootstrapped confidence intervals (BCa method with 2000 samples without stratification) tested associations between self‐reported EG (RDEES differentiation) and the SI for negative and positive trials. For correlation analyses, we applied Bonferroni correction across six tests (adjusted significance for six comparisons: *p* < 0.0083). Associations between EG (Level 2: RDEES differentiation and specificity indices for negative and positive videos) and total fixation duration as well as number of revisits (dependent variables) were investigated via multilevel‐models (MLMs) with random intercepts across participants [i.e., Dependent Variable ∼ 1 + EG + (1|participant)]. For MLMs, we defined two outcome families (Total Fixation Duration Percentage and Number of Revisits) each evaluated in two conditions (negative and positive videos). We controlled the familywise error rate with a Bonferroni correction within each family (i.e., across two tests: adjusted significance for two comparisons: *p* < 0.025). MLMs were calculated using the *General Analyses for Linear Models* (*GAMLj*) package (version: 3.6.1) in *JAMOVI* (version: 2.7). Anonymized JAMOVI files are available in the Open Science Framework (OSF) at https://osf.io/ra7ku/.

## 3 Results

### 3.1 Manipulation Check

After positive trials, valence (*M* = 7.46, SD = 0.91) was higher than after negative trials [*M* = 5.32, SD = 1.70; *t*(150) = 16.50, *p* < 0.001]. Unlike in the previous study (Potthoff et al. 2023), average valence after negative trials was not below the neutral midpoint (i.e., 5). Therefore, we calculated all analyses involving negative trials, once without excluding participants (as preregistered) and additionally once after excluding participants with an average valence of mood above 5 after negative trials (Supporting Information S1).

### 3.2 Intercorrelations Emotional Granularity (Specificity Index & Self‐Reported)

The SI for negative trials (*M* = 75%, SD = 19%) was not correlated with self‐reported EG [RDEES differentiation; *M* = 3.52, SD = 0.87; *r*(149) = 0.097, *p* = 0.235]. Also, the correlation between the SI for positive trials (*M* = 60%, SD = 21%) and RDEES differentiation (*M* = 3.52, SD = 0.87) was not statistically significant [*r*(149) = 0.040, *p* = 0.628]. For all correlations between EG and gaze behavior as well as for confidence intervals, see Table 1 (negative trials) and Table 2 (positive trials).

**TABLE 1:**
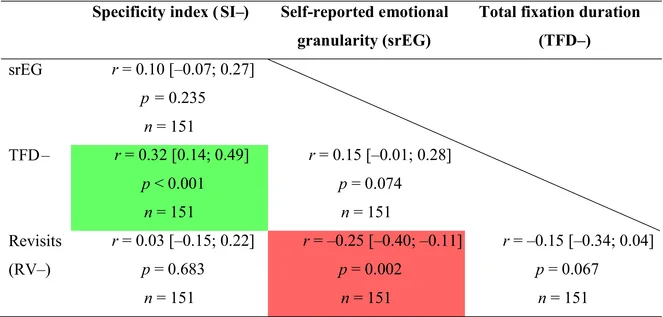
Correlations between emotional granularity & gaze behavior.

*Note:* Pearson correlations between Specificity Index for negative trials (SI−), self‐reported Emotional Granularity (srEG), total fixation duration percentage on negative videos (TFD−), and revisits on negative videos (RV−). 95% BCa confidence intervals: [lower bound; upper bound]. Bootstrapping was conducted without stratification by participant. Significant (adjusted for six comparisons: *p* < 0.0083) positive correlations highlighted green. Significant negative correlations highlighted red.

**TABLE 2:**
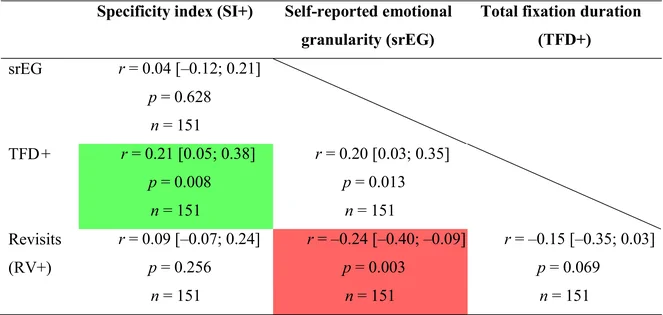
Correlations between emotional granularity & gaze behavior. Positive trials.

*Note:* Pearson correlations between Specificity Index for positive trials (SI+), self‐reported Emotional Granularity (srEG), total fixation duration percentage on positive videos (TFD+), and revisits on positive videos (RV+). 95% BCa confidence intervals: [lower bound; upper bound]. Bootstrapping was conducted without stratification by participant. Significant (adjusted for six comparisons: *p* < 0.0083) positive correlations highlighted green. Significant negative correlations highlighted red.

### 3.3 Multilevel Models Total Fixation Duration Percentage

#### 3.3.1 Negative Videos

The specificity index for negative videos, *t*(148.9) = 4.17, *p* < 0.001, *B* = 0.248, 95% CI [0.132; 0.365], SE = 0.059, but not self‐reported EG (i.e., RDEES differentiation, srEG), *t*(149.1) = 1.80, *p* = 0.074, *B* = 0.025, 95% CI [−0.002; 0.052], SE = 0.014, was positively associated with the total fixation duration percentage on negative videos (*M* = 76%, SD = 15%). For the full MLM model as well as bootstrapped confidence intervals, see Table 3.

**TABLE 3:** Results of the multilevel models for the specificity index (SI) and self‐reported emotional granularity (srEG) predicting total fixation duration percentage on negative and positive videos.

	Negative		Positive	
*Effect*	*B* [bootstrapped 95% Cis with 5000 iterations]			
Intercept	0.58*** [0.49–0.66]	0.68*** [0.58–0.77]	0.65*** [0.58–0.71]	0.62*** [0.52–0.71]
SI	0.25*** [0.14–0.37]		0.15** [0.04–0.26]	
srEG		0.02 [−0.00–0.05]		0.03* [0.01–0.06]
*Random effects*				
*σ* ^2^	0.02	0.02	0.03	0.03
*τ*_00_	0.02_participant_	0.02_participant_	0.02_participant_	0.02_participant_
ICC	0.39	0.41	0.36	0.36
*N*	151_participant_	151_participant_	151_participant_	151_participant_
Observations	903	903	904	904
Marginal *R* ^2^	0.054	0.011	0.021	0.019
Conditional *R* ^2^	0.421	0.421	0.371	0.371

*Note:* This table was created with siPlot (Lüdecke 2025).

Abbreviations: SI, specificity index; srEG, self‐reported emotional granularity.

* *p* < 0.05.

** *p* < 0.01.

*** *p* < 0.001.

#### 3.3.2 Positive Videos

For positive trials, there was a positive association between the specificity index and total fixation duration percentage (*M* = 74%, SD = 15%), *t*(149.4) = 2.66, *p* = 0.009, *B* = 0.150, 95% CI [0.039; 0.260], SE = 0.056, as well as between self‐reported EG and total fixation duration percentage, *t*(149.1) = 2.49, *p* = 0.014, *B* = 0.034, 95% CI [0.007; 0.061], SE = 0.014. For the full MLM model as well as bootstrapped confidence intervals, see Table 3.

### 3.4 Multilevel Models Number of Revisits

#### 3.4.1 Negative Videos

Revisits of negative videos (*M* = 0.94, SD = 0.53) were not significantly associated with the specificity index, *t*(149.0) = −1.08, *p* = 0.281, *B* = −0.246, 95% CI [−0.692; 0.200], SE = 0.227. However, self‐reported EG was negatively associated with revisits on negative videos, *t*(149.0) = −2.74, *p* = 0.007, *B* = −0.134, 95% CI [−0.230; −0.038], SE = 0.049. For the full MLM model as well as bootstrapped confidence intervals, see Table 4.

**TABLE 4:** Results of the multilevel models for the specificity index (SI) and self‐reported emotional granularity (srEG) predicting revisits on negative and positive videos.

	Negative		Positive	
*Effect*	*B* [bootstrapped 95% Cis with 5000 iterations]			
Intercept	1.06*** [0.47–1.74]	1.36*** [0.76–1.98]	0.81*** [0.36–1.27]	1.49*** [0.82–2.11]
SI	−0.24 [−1.16–0.59]		0.24 [−0.45–1.04]	
srEG		−0.13** [−0.30–0.04]		−0.15** [−0.32–0.02]
*Random effects*				
*σ* ^2^	0.53	0.53	0.63	0.63
*τ*_00_	0.19_participant_	0.18_participant_	0.20_participant_	0.18_participant_
ICC	0.27	0.26	0.24	0.22
*N*	151_participant_	151_participant_	151_participant_	151_participant_
Observations	906	906	906	906
Marginal *R* ^2^	0.003	0.019	0.003	0.021
Conditional *R* ^2^	0.270	0.270	0.241	0.240

*Note:* This table was created with siPlot (Lüdecke 2025).

Abbreviations: SI, specificity index; srEG, self‐reported emotional granularity.

* *p* < 0.05.

** *p* < 0.01.

*** *p* < 0.001.

#### 3.4.2 Positive Videos

Also for positive trials only self‐reported EG, *t*(149.0) = −3.04, *p* = 0.003, *B* = −0.153, 95% CI [−0.253; −0.054], SE = 0.050, but not the specificity index, *t*(149.0) = 1.14, *p* = 0.256, *B* = 0.242, 95% CI [−0.175; 0.661], SE = 0.213, was negatively associated with the number of revisits (*M* = 0.95, SD = 0.55). For the full MLM model as well as bootstrapped confidence intervals, see Table 4.

### 3.5 Exploratory Verbal Fluency Analyses

Verbal fluency (Tombaugh 1999) did not correlate significantly with the SI after negative trials and positive trials [negative: *r*(149) = −0.017, *p* = 0.833; positive: *r*(149) = −0.087, *p* = 0.286]. Also, the correlation between verbal fluency and self‐reported EG was not significant [*r*(149) = 0.040, *p* = 0.624].

## 4 Discussion

The present study replicated and extended the work of Potthoff et al. (2023) by examining associations between EG and visual attention to emotional stimuli in a more complex and, therefore, more ecologically valid context. Specifically, we investigated whether performance‐based EG (Ottenstein and Lischetzke 2020) and self‐reported EG (Kang and Shaver 2004) predicted gaze behavior when affective videos (Blechert et al. 2013) were presented alongside dynamic distractors. Our findings complement previous research with static pictorial distractors, which analyzed how different forms of EG are linked to visual attention in emotional contexts (Potthoff et al. 2023).

### 4.1 Associations Gaze Behavior and Emotional Granularity

Higher performance‐based EG, measured by the SI (Ottenstein and Lischetzke 2020), was associated with increased fixation duration on both negative and positive emotional videos. Participants who described their emotions with greater specificity sustained their visual attention on affective stimuli, potentially indicating greater engagement or more thorough processing of affect elicitors. This association between total fixation duration and EG was not observed in the replicated study (Potthoff et al. 2023), which may be due to their use of static and potentially less engaging distractor stimuli. In the previous study, participants may have found the distractors less interesting regardless of their motivation to engage with the affective content. In the present study, the distractors may have been a more appealing alternative, drawing attention away from the emotional videos for participants less motivated to engage with the affective videos. Furthermore, contrary to our expectations and previous findings by Potthoff et al. (2023), the number of revisits to emotional videos was not associated with the SI. However, self‐reported EG (RDEES differentiation; Kang and Shaver 2004) was negatively associated with revisits to negative and positive emotional stimuli. In other words, participants who reported greater awareness of subtle differences in their affective experiences were less likely to repeatedly return their gaze to emotional stimuli. This pattern may indicate that self‐perceived affect differentiation abilities reduce the subjective need for re‐evaluating the elicitor of affect.

### 4.2 Self‐Reported and Performance‐Based Emotional Granularity

The lack of correlation between the SI and self‐reported EG is only somewhat surprising. The replicated study (Potthoff et al. 2023) found a small association between these two EG measures only in the context of negative affect, but not for positive affect trials. Similarly, when developing the SI, Ottenstein and Lischetzke (2020) also reported no significant association and Wabnegger et al. (2024) also found no correlation between self‐reported and performance‐based EG. This lack of a (strong) link between the two EG measures underscores fundamental differences between the two approaches of assessing EG. Self‐reported EG may be biased by beliefs about oneself and self‐perceptions, rather than reflecting solely the actual emotional differentiation ability (MacCann et al. 2003; Robinson and Clore 2002). Furthermore, self‐reported EG depends on the ability to recall emotional situations accurately, while performance‐based EG, like the SI, is assessed immediately after the affect was elicited (like in the present study) or shortly after (e.g., in ecological momentary assessments; compare Wabnegger et al. 2024). Additionally to such memory biases, self‐reported EG may be affected by a self‐concept bias to a larger degree than performance‐based EG. Such a self‐concept bias may serve to protect the existing self‐concept about emotion differentiation abilities of people (Wabnegger and Schienle 2025). The present findings add to the null findings on associations between performance‐based and self‐reported EG which suggest that perceiving oneself as capable of distinguishing subtle emotional nuances does not necessarily correspond to using this perceived ability when describing one's own affective state (i.e., higher performance‐based EG). This divergence between self‐reported and performance‐based EG also suggests opportunities for interventions. People with high self‐reported EG but low performance‐based EG might benefit from trainings designed to enhance their ability to apply emotional differentiation. If people report to differentiate between different nuances of affect even though they actually do so less than others, emotion differentiation trainings could help bridge the gap between perceived and actual emotional differentiation abilities.

The positive association between performance‐based EG and total fixation duration suggests that sustained engagement with affect‐eliciting stimuli may support a more nuanced differentiation of emotional experiences. This finding aligns with the assumption that people with higher EG are more likely to use adaptive regulation strategies that involve confronting and processing emotions (i.e., attending to and engaging with elicitors of emotions) rather than avoiding them (Barrett et al. 2001).

In contrast, the negative association between self‐reported EG and the number of revisits may reflect a subjectively perceived lower need to re‐evaluate affective information. People who perceive themselves as having high EG may feel confident in their initial assessments and, therefore, see less need to redirect attention repeatedly toward emotional stimuli. It is possible that this association was not observed in the replicated study (Potthoff et al. 2023) due to the use of static and potentially less engaging distractors, which may have captured participants' attention to a similar extent regardless of their level of self‐reported EG.

### 4.3 Methodological Considerations

The different findings compared to the replicated study (Potthoff et al. 2023) can be attributed to methodological differences. A key methodological advance of this study compared to the replicated one was the use of dynamic distractors, which increased ecological validity. The divergence from previous findings concerning total fixation duration and revisits may be attributable to this change. It is possible that static distractors in prior studies did not provide a sufficiently compelling alternative focus of attention, thus inflating exploratory revisits to emotional stimuli among people with higher performance‐based EG. The current design more closely mimics real‐world visual environments, where emotional information competes with other engaging and dynamic visual stimuli.

### 4.4 Limitations and Future Directions

Several limitations have to be considered. First, the German speaking participants were primarily university students (i.e., young and highly educated), limiting the generalizability of the findings: How emotions are described may be affected by various demographic data and between languages. Second, despite improving ecological validity, the emotional impact of brief video feedback may still be limited compared to real‐life affective interactions. The participants did not receive valid feedback on an actually performed task. Future work could incorporate more immersive or personally relevant stimuli (e.g., actual face‐to‐face interactions) to examine how EG influences emotion processing and visual attention in more ecologically valid contexts.

Finally, a longitudinal or experimental design could test whether EG variation over time or training in emotion differentiation leads to changes in attention allocation and emotion regulation over time.

### 4.5 Conclusion

Our findings suggest that different measures of EG are uniquely associated with visual attention to emotional stimuli in complex environments: While performance‐based EG predicted sustained attention to affective content, self‐reported EG was linked to reduced re‐evaluation of positive stimuli which may indicate a lower perceived need to gather (further) affective information. These results emphasize the importance of distinguishing between different forms of EG and highlight how attention during emotion processing may vary depending on the complexity of context.

## Author Contributions

**Jonas Potthoff:** conceptualization, methodology, validation, formal analysis, investigation, data curation, writing – original draft, writing – review and editing, visualization, supervision, project administration. **Albert Wabnegger:** conceptualization, methodology, validation, writing – review and editing. **Anne Schienle:** conceptualization, methodology, validation, writing – review and editing, supervision, project administration.

## Funding

The authors have nothing to report.

## Disclosure

*Material availability statement*: The affect eliciting videos (E.Vids; Blechert et al. 2013) cannot be provided openly but the full video set can be requested from the authors (https://www.eat.sbg.ac.at/resources/emovid). In the present study, we used videos with the actors 10, 11, 12, 13, 14, 15, 16, 17, 20, 21, 22, and 23. The twelve distractor videos were uploaded to the Distractor Videos folder in the OSF project (https://osf.io/je3ar/?view_only=33f10b10810c47d59fc95e2eb90dc986).

## Ethics Statement

The study received approval from the university's ethics committee (ethics approval code: GZ. 39/69/63 ex 2022/23) and was conducted in accordance with the Declaration of Helsinki.

## Consent

All participants provided informed consent before participating in the experiment.

## Conflicts of Interest

The authors declare no conflicts of interest.

## Supporting information


**Supporting Information: S1** Reduced sample results for negative trials.
**Table S1:** Correlations between emotional granularity & gaze behavior. Negative trials.
**Table S2:** Means and Standard Deviations for Self‐report Data, Specificity Indices and Gaze Data.

## Data Availability

The OSF‐project https://osf.io/ra7ku/ contains two jamovi files which document the analyses reported in this manuscript (OpenDataLong_for3point3_3point4.omv for MLM analyses from Sections 3.3 and 3.4 & OpenDataWide_for_3point1_3point2.omv for correlation analyses from Sections 3.1 and 3.2). We also provide a list of adjectives with specificity ratings of all three raters and inter rater reliability calculations (adjective_list.omv) as well as a file containing gaze data on trial level and questionnaire data on item level (OpenDataWide_TrialLevel_eyeTracking_and_QuestionnaireItems.omv).

## Endnotes

^1^
Videos of the following actors from the *E.Vids* set were used: 10, 11, 12, 13, 14, 15, 16, 17, 20, 21, 22 and 23.
