# Supplementary material for: Another Differentiated Look at Emotions: A Replication Study on Associations Between Gaze Behavior During the Processing of Affective Videos and Emotional Granularity
Source: Scand J Psychol. 2026 May 15;67(6):1373–81. doi: 10.1111/sjop.70112 (PMC13739009; doi:10.1111/sjop.70112)
Supplement: Supplementary file 1 [file SJOP-67-1373-s001.docx]

**Supplementary Material**

**S1. Reduced Sample Results for Negative Trials**

**S1.1. Intercorrelations Emotional Granularity (Specificity Index & Self-Reported)**

Corresponds with section 3.2. in the manuscript: The SI for negative trials (*M* = 77%, *SD* = 19%) was not correlated with self-reported EG [RDEES differentiation; *M* = 3.42, *SD* = 0.80; *r*(68) = .077, *p* = .527]. For all correlations between EG and gaze behaviour as well as for confidence intervals, see Supplementary Table 1.

**Supplementary Table 1:** Correlations Between Emotional Granularity & Gaze Behaviour. Negative Trials

|  | **Specificity Index (SI-)** | **Self-reported Emotional Granularity (srEG)** | **Total Fixation Duration (TFD-)** |
| --- | --- | --- | --- |
| **srEG** | *r* = .08 [-.14; .31]  *p* = .527  *n* = 70 |  |  |
| **TFD-** | *r* = .36 [.05; .61]  *p* = .002  *n* = 70 | *r* = .07 [-.14; .28]  *p* = .567  *n* = 70 |  |
| **Revisits (RV-)** | *r* = .23 [-.01; .40]  *p* = .061  *n* = 70 | *r* = -.21 [-.38; -.02]  *p* = .081  *n* = 70 | *r* = -.16 [-.40; .09]  *p* = .186  *n* = 70 |

*Note:* Pearson correlations between Specificity Index for negative trials (SI-), self-reported Emotional Granularity (srEG), total fixation duration percentage on negative videos (TFD-), and revisits on negative videos (RV-). 95% BCa confidence intervals: [lower bound; upper bound]. Bootstrapping was conducted without stratification by participant. Significant (adjusted for six comparisons: *p* < .0083) positive correlations highlighted green. Supplementary Table 1 corresponds with Table 1 in the manuscript.

**S1.2. Multilevel Models Total Fixation Duration Percentage for Negative Videos**

Corresponds with section 3.3.1. in the manuscript: The specificity index for negative videos, *t*(67.9) = 3.15, *p* = .002, B = 0.291, 95% CI [0.109; 0.471], SE = 0.092, but not self-reported EG (i.e., RDEES differentiation), *t*(68.0) = 0.58, *p* = .562, B = 0.013, 95% CI [−0.032; 0.059], SE = 0.023 was positively associated with the total fixation duration percentage on negative videos (*M* = 78%, *SD* = 15%).

**S1.3. Multilevel Models Number of Revisits of Negative Videos**

Corresponds with section 3.4.1. in the manuscript: Revisits of negative videos (*M* = 0.91, *SD* = 0.51) were not significantly associated with the specificity index, *t*(68.0) = 1.32, *p* = .190, *B* = 0.402, 95% CI [-0.194; 0.998], *SE* = 0.303., or self-reported EG, *t*(68.0) = -1.14, *p* = .258, *B* = -0.081, 95% CI [-0.222; 0.589], *SE* = 0.071.

**S2. Descriptive Statistics**

**S2.1. Supplementary Table 2**

|  | **Full Sample**  **(n = 151)** | **Reduced Sample**  **(n = 70)** |
| --- | --- | --- |
| **Valence of Mood after Negative Trials** | *M* = 5.32  *SD* = 1.70 | *M* = 3.78  *SD* = 0.86 |
| **Valence of Mood after Positive Trials** | *M* = 7.46  *SD* = 0.91 | *M* = 7.15  *SD* = 0.85 |
| **Specificity Index for Negative Trials**  **(SI-)** | *M* = .75  *SD* = .19 | *M* = .77  *SD* = .19 |
| **Specificity Index for Positive Trials**  **(SI+)** | *M* = .60  *SD* = .21 | *M* = .62  *SD* = .20 |
| **Self-reported Emotional Granularity**  **(srEG)** | *M* = 3.52  *SD* = 0.87 | *M* = 3.42  *SD* = 0.80 |
| **Total Fixation Duration on Negative Videos**  **(TFD-)** | *M* = 76.3%  *SD* = 14.6% | *M* = 77.7%  *SD* = 15.3% |
| **Total Fixation Duration on Positive Videos**  **(TFD+)** | *M* = 73.5%  *SD* = 14.8% | *M* = 76.3%  *SD* = 14.3% |
| **Revisits of Negative Videos**  **(RV-)** | *M* = 0.94  *SD* = 0.53 | *M* = 0.91  *SD* = 0.51 |
| **Revisits of Positive Videos**  **(RV+)** | *M* = 0.95  *SD* = 0.55 | *M* = 0.93  *SD* = 0.56 |

*Note:* Means and standard deviations for Valence of mood after negative and positive trials (from 1 to 9), specificity index for negative (SI-) and positive (SI+) trials (from 0 to 1), self-reported emotional granularity (srEG: differentiation scale of the Range and Differentiation of Emotional Experience Scale: RDEES; Kang & Shaver, 2004), total fixation duration on negative (TFD-) and positive (TFD+) videos (in %), and number of revisits of negative (RV-) and positive (RV-) videos for the whole sample (n = 151) as well as the reduced sample (n = 70) with participants excluded, who reported an average valence of mood above 5 after negative trials.
